# NTN4 as a prognostic marker and a hallmark for immune infiltration in breast cancer

**DOI:** 10.1038/s41598-022-14575-2

**Published:** 2022-06-22

**Authors:** Lili Yi, Yongqiang Lei, Fengjiao Yuan, Conghui Tian, Jian Chai, Mingliang Gu

**Affiliations:** Joint Laboratory for Translational Medicine Research, Liaocheng People’s Hospital, Liaocheng, 252000 China

**Keywords:** Breast cancer, Prognostic markers

## Abstract

Netrin-4 (NTN4), a member of neurite guidance factor family, can promote neurite growth and elongation. This study aims to investigate if NTN4 correlates with prognosis and immune infiltration in breast cancer. The prognostic landscape of NTN4 and its relationship with immune infiltration in breast cancer were deciphered with public databases and immunohistochemistry (IHC) in tissue samples. The expression profiling and prognostic value of NTN4 were explored using UALCAN, TIMER, Kaplan–Meier Plotter and Prognoscan databases. Based on TIMER, relationships of NTN4 expression with tumor immune invasion and immune cell surface markers were evaluated. Transcription and survival analyses of NTN4 in breast cancer were investigated with cBioPortal database. The STRING database was explored to identify molecular functions and signaling pathways downstream of NTN4. NTN4 expression was significantly lower in invasive breast carcinoma compared with adjacent non-malignant tissues. Promoter methylation of NTN4 exhibited different patterns in breast cancer. Low expression of NTN4 was associated with poorer survival. NTN4 was significantly positively related to infiltration of CD8^+^ T cells, macrophages and neutrophils, whereas significantly negatively related to B cells and tumor purity. Association patterns varied with different subtypes. Various associations between NTN4 levels and immune cell surface markers were revealed. Different subtypes of breast cancer carried different genetic alterations. Mechanistically, NTN4 was involved in mediating multiple biological processes including morphogenesis and migration.

## Introduction

Netrins belong to a conserved laminin-like secreted protein family, originally identified as axon-guiding molecules^1^. Netrins are expressed in ectopic nervous system, involved in a variety of biological processes, including tissue morphogenesis^2^, angiogenesis^3^, lymphangiogenesis^4^, tumorigenesis^5^, migration^6^, invasion^7^, adhesion^8^, apoptosis^9^ and inflammation^10^. Netrins are highly conserved during evolution. Netrin1 (NTN1), netrin3 (NTN3) and netrin4 (NTN4) have been identified in mammals. Netrin-4 (NTN4, also known as β-netrin) is a new member of Netrins family in vertebrates, localized to the basement membrane surrounding lobular structures in the blood vessels, kidneys, breasts and ovaries^11^. NTN4 is secreted by breast epithelial cells and sequestered by the basement membrane. NTN4 was highly expressed in invasive breast adenocarcinoma^12^. NTN4 may participate in the development and progression of a variety of cancers, NTN4 was proposed to serve as a prognostic biomarker for breast cancer^13,14^. Based on these findings, we aim to explore if NTN4 affects the prognosis of breast cancer patients. Furthermore, no investigation has been focused on the relationship of NTN4 with tumor microenvironment (TME) of breast cancer. Whether NTN4 expression is associated with immune infiltration in the TME or clinical outcome remains undetermined.

In early 2021, the World Health Organization (WHO) International Agency for Research on Cancer agency (IARC) published 2020’s global cancer data (https://www.iarc.fr/faq/latest-global-cancer-data-2020-qa/). Breast cancer (BC) has replaced lung cancer as the most common malignancy globally, with an estimated 2.26 million cases annually worldwide, which ranks the first in both morbidity and mortality among women. Breast cancer is a serious threat to human health. Continuous development of molecular markers specific to celluar subsets and targeted therapies^15^ will become an important research direction in the future. In recent decades, prognostic predictive value of mRNA expression has become increasingly attractive. Transcriptome of primary breast tumors can help predict intrinsic subtypes, tumor grades, drug response, risk of recurrence, and survival^16–18^.

Here, for the first time, we have provided supporting evidence for relationship between NTN4 and immune infiltration as well as clinical outcome. In this study, association between NTN4 mRNA and breast cancer prognosis was evaluated by using public databases such as Kaplan–Meier plotter and PrognoScan. In addition, associations of NTN4 mRNA levels with clinicopathological characteristics and tumor-infiltrating immune cells were investigated in breast cancer. Meanwhile, gene alterations, molecular functions and regulation pathways of NTN4 were explored. Our findings shed light on the role of NTN4 in breast cancer and provided potential interaction between NTN4 and the TME.

## Materials and methods

### Tissue samples

Three pairs of tissue from breast cancer patients were analyzed. The criteria for tissue included an original histological diagnosis of invasive breast carcinoma, and the efficiency of clinical pathological data. Specimens were frozen in liquid nitrogen (− 80 °C) for analysis. The study was conducted in accordance with the Declaration of Helsinki and was approved by The Ethical Committee of Liaocheng People’s Hospital and each patient provided informed consent. This study was performed according to the REMARK guidelines.

### TIMER database analysis

We analyzed relationship of NTN4 gene with respective abundance of infiltrating immune cells (B cells, CD4^+^ T cells, CD8^+^ T cells, neutrophils, macrophages, and dendritic cells (DC)) in breast cancer patients using The Tumor IMmune Estimation Resource (TIMER) algorithm database (https://cistrome.shinyapps.io/timer/)^19^. Tumor purity is a vital factor that influences immune infiltration in tumor samples by genomic approaches.

### RNA-sequencing data and bioinformatics analysis

NTN4 was preliminarily proposed as a prognostic marker for breast cancer patients, however, it has not been verified in the cohorts with large sample sizes. This is the first study to confirm the clinical significance of NTN4 in breast cancer in large cohorts. METABRIC database accurately analyzed breast cancer subtypes. The Cancer Genome Atlas (TCGA) database was applied to collect RNA-seq data and clinical information from 1098 cases of breast cancer. The original format of downloaded data was level 3 HTSeq-fragments per kilobase per million (FPKM) and converted into transcripts per million (TPM) for subsequent analysis. Paired and unpaired tests were performed to compare expression patterns of NTN4. Area under curve (AUC) of NTN4 was analyzed, to determine whether NTN4 can be used as a biomarker to distinguish between tumor and adjacent tissues. These analyses were conducted with R software.

### UALCAN database analysis

The UALCAN database (ualcan.path.uab.edu/index.html) was applied to analyze relationships of NTN4 mRNA expression or NTN4 promoter methylation levels with clinicopathological characteristics^20^.

### Survival Analysis using PrognoScan and Kaplan–Meier Plotter

To investigate the prognostic value of NTN4 mRNA in breast cancer, Kaplan–Meier Plotter (http://www.kmplot.com, P-value < 0.05)^21^ and PrognoScan database^22^ (http://dna00.bio.kyutech.ac.jp/PrognoScan/, adjust the threshold Cox P-value < 0.05) were applied. Specifically, NTN4 expression level was searched in all available microarray datasets of PrognoScan to determine its relationship with prognosis. We selected four datasets for analyzing NTN4 expression in breast cancer. The threshold was set as a Cox P-value < 0.05.

### Gene alterations of NTN4 in breast cancer using cBioPortal

Gene alteration of NTN4 was explored using the cBioPortal (http://www.cbioportal.org) regarding BC. We selected Breast Invasive Carcinoma (TCGA, PanCancer Atlas) that contains 1084 samples to subsequent analyses. OncoPrint was constructed in the cBioPortal (TCGA provisional) to directly reflect all types of changes in NTN4 gene amplification, deep deletion, mRNA upregulation, and mRNA downregulation in patients with BC. In addition, potential effects of NTN4 gene alterations on survival of BC patients were estimated using Kaplan–Meier survival curves in the cBioPortal.

### STRING database

For an in-depth exploration of relationship, the STRING database (version 11.0) was applied (https://cn.string-db.org/cgi/network?taskId=bsL1tXI2yNb4&sessionId=b7FzfW5U0kqB)^23^. The STRING contains both known and predicted protein–protein associations based on bioinformatic resources, including curated databases, experimental/biochemical data, PubMed abstracts, and others. Using the NTN4 as an input parameter, the proteins that might interact with NTN4 were searched. The default scoring threshold of interaction was 0.4, and a subnetwork constructed with genes that might interact with NTN4 was extracted. The NTN4 driving genes and interactive genes were constructed into a network. Then, the STRING database was used to conduct gene ontology (GO) enrichment and Kyoto Encyclopedia of Genes and Genomes (KEGG) pathway analyses of all selected genes.

### Immunohistochemistry (IHC)

The expression of NTN4 protein was verified by IHC. Tissue paraffin sections were dewaxed in xylene (Yantai fast eastern fine chemical CO., LTD) for three times, each for 10 min. Sections were followed by serial rehydration in graded ethanol (Yantai fast eastern fine chemical CO., LTD) from 100% ethanol followed by 95%, 90%, 80%, 70% and 60% ethanol, and finally in distilled water. Heat-mediated antigen retrieval was conducted in Ethylene Diamine Tetraacetic Acid (EDTA) buffer (pH 9.0) (MVS-0098; MXB) using a microwave pressure cooker for 10 min. They were blocked with 5% Bovine Serum Albumin (BSA) for 30 min at 37 °C, followed a mouse anti-NTN4 monoclonal antibody (sc-365280; Santa Cruz Biotechnology) at 1:100 dilution for the night at 4 °C. Sections were washed three times by Phosphate Buffer Saline (PBS) (pH 7.2) for 5 min. Binding of the anti-NTN4 antibody was detected using Biotin-conjugated secondary anti-mouse antibody from BOSTER detection system (SA1051) for 30 min at 37 °C, followed washed 3 times by PBS (pH 7.2) for 5 min. Next, Sections were incubated with SABC-AP (SA1051; BOSTER) for 30 min at 37 °C, followed washed four times by 0.01 M Tris Buffer Saline (TBS) (pH 9.0–9.5) for 5 min. And they developed with BCIP/NBT as the chromogen for 30 min. The sections were counterstained with Nuclear fast red (SA1051; BOSTER) for 5 min. For the determination of the NTN4 expression, three pathologists used a double-blind method to randomly select 3–5 high-power visual fields in order to determine the staining intensity and staining positive rate. Staining intensity score was defined as follows: 0 (negative), 1 (weak positive), 2 (moderate positive) and 3 (strong positive). Positive rate score was defined as follows: 0 (0%), 1 (1–25%), 2 (26–50%), 3 (51–75%), 4 (76–100%). Cumulative score = staining intensity × staining distribution. The cumulative score < 4 was considered as low NTN4 expression, whereas ≥ 4 as high NTN4 expression.

### Statistical analysis

Wilcoxon test was used to compare different expression levels of NTN4 in different cancers. Kaplan–Meier plot was used to estimate survival curve. In order to describe survival curve more accurately, log rank test was used to calculate log rank P value. Univariate Cox regression model was applied to calculate hazard ratio (HR), 95% confidence intervals (CI) and Cox P values in PrognoScan. Spearman’s coefficient was used to analyze the correlation of gene expression. Using receiver operating characteristic (ROC) curve of NTN4, optimal cut-off point was calculated to distinguish “high” from “low” expression, and a ROC was generated with MedCalc in R version 4.0.2 (https://www.r-project.org/). In the absence of special circumstances, a P < 0.05 was considered statistically significant.

## Results

### The mRNA expression and protein expression of NTN4

**Figure 1:**
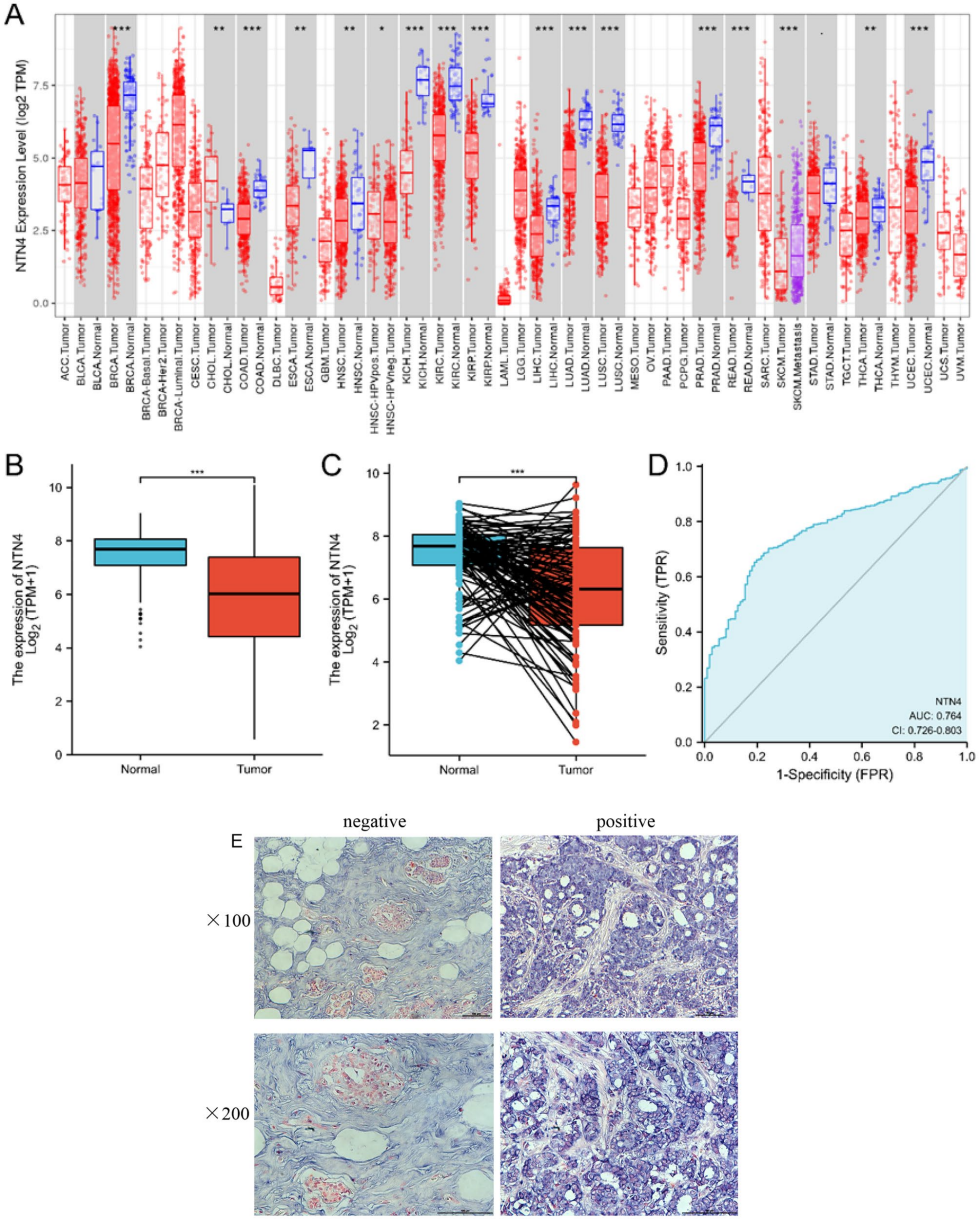
NTN4 expression and clinicopathological features of breast cancer. (**A**) NTN4 mRNA expression levels in different tumor types from TCGA database were determined by TIMER (*P < 0.05, **P < 0.01, ***P < 0.001). (**B**) Wilcoxon rank sum test was used to analyze differential expression of NTN4 between BC tumor and adjacent tissue. (**C**) Wilcoxon signed rank sum test was used to detect the differential expression of NTN4 between BC tumor and adjacent tissue. (**D**) ROC curve established efficiency of NTN4 mRNA expression level on distinguishing BC tumor from non-tumor tissue. X-axis represents false positive rate, and Y-axis represents true positive rate. (E): Representative negative (adjacent tissue) and positive (breast tumor tissue) expression of NTN4 protein as demonstrated by IHC. The magnification is 100 × (upper) or 200 × (lower), respectively.

To evaluate NTN4 mRNA expression in pan-cancer, RNA sequencing data in TCGA was examined using TIMER. The differential NTN4 mRNA expression patterns between tumorous and adjacent tissues were summarized in Fig. 1A. NTN4 mRNA expression was significantly lower in invasive breast carcinoma (BRCA), as well as in basal, human epidermal growth factor receptor 2 (Her2^+^), and luminal breast cancer subtypes, compared with adjacent tissues. Meanwhile, expression of NTN4 in tumor was significantly lower than those in adjacent tissue in unpaired (Fig. 1B) and paired samples (Fig. 1C). In addition, ROC curve was used to analyze effectiveness of NTN4 mRNA expression level AUC on distinguishing breast cancer tissues from non-tumor tissues. The AUC of NTN4 was 0.764, suggesting that NTN4 could serve as a biomarker to distinguish BC from non-tumor tissue (Fig. 1D). NTN4 protein expression was verified by IHC in adjacent tissue and tumor tissue (Fig. 1E).

Demographic and clinical characteristics of patients were summarized in Table 1, in which 1083 primary breast cancer cases were collected from TCGA database. According to relative NTN4 levels, breast cancer patients were divided into low (n = 541) and high (n = 542) expression groups. The associations between NTN4 expression levels and clinicopathological characteristics were evaluated. Chi-square tests revealed that NTN4 expression was associated with T stage (P < 0.001), Histological type (P < 0.001), Pathologic stage (P = 0.004), progesterone receptor (PR) and estrogen receptor (ER) status (P < 0.001). No significant correlation was observed between NTN4 expression and age (P = 0.035), M stage (P = 1.000), menopausal status (P = 0.916) or HER2 status (P = 0.438).

**Table 1:** The relationship between the expression of NTN4 and clinicopathological data.

Characteristic	NTN4 mRNA		P
	Low(n = 541)	High(n = 542)	
**Age (years)**			0.035
Median (IQR)	57 (48, 66)	60 (49, 68)	0.012
< = 60	318 (29.4%)	283 (26.1%)	
> 60	223 (20.6%)	259 (23.9%)	
**T stage, n (%)**			< 0.001
T1	113 (10.5%)	164 (15.2%)	
T2	349 (32.3%)	280 (25.9%)	
T3	60 (5.6%)	79 (7.3%)	
T4	17 (1.6%)	18 (1.7%)	
**N stage, n (%)**			0.653
N0	258 (24.2%)	256 (24.1%)	
N1	179 (16.8%)	179 (16.8%)	
N2	61 (5.7%)	55 (5.2%)	
N3	33 (3.1%)	43 (4%)	
**M stage, n (%)**			1.000
M0	452 (49%)	450 (48.8%)	
M1	10 (1.1%)	10 (1.1%)	
**Menopause status, n (%)**			0.916
Pre	117 (12%)	112 (11.5%)	
Peri	20 (2.1%)	20 (2.1%)	
Post	348 (35.8%)	355 (36.5%)	
**Histological type, n (%)**			< 0.001
Infiltrating Ductal Carcinoma	440 (45%)	332 (34%)	
Infiltrating Lobular Carcinoma	52 (5.3%)	153 (15.7%)	
**Pathologic stage, n (%)**			0.004
Stage I	71 (6.7%)	110 (10.4%)	
Stage II	335 (31.6%)	284 (26.8%)	
Stage III	117 (11%)	125 (11.8%)	
Stage IV	8 (0.8%)	10 (0.9%)	
**PR status, n (%)**			< 0.001
Negative	267 (25.8%)	75 (7.3%)	
Indeterminate	3 (0.3%)	1 (0.1%)	
Positive	245 (23.7%)	443 (42.8%)	
**ER status, n (%)**			< 0.001
Negative	202 (19.5%)	38 (3.7%)	
Indeterminate	1 (0.1%)	1 (0.1%)	
Positive	313 (30.2%)	480 (46.4%)	
**HER2 status, n (%)**			0.438
Negative	264 (36.3%)	294 (40.4%)	
Indeterminate	7 (1%)	5 (0.7%)	
Positive	82 (11.3%)	75 (10.3%)	

*IQR* interquartile range.

### Relationships between NTN4 mRNA expression and clinicopathological characteristics

**Figure 2:**
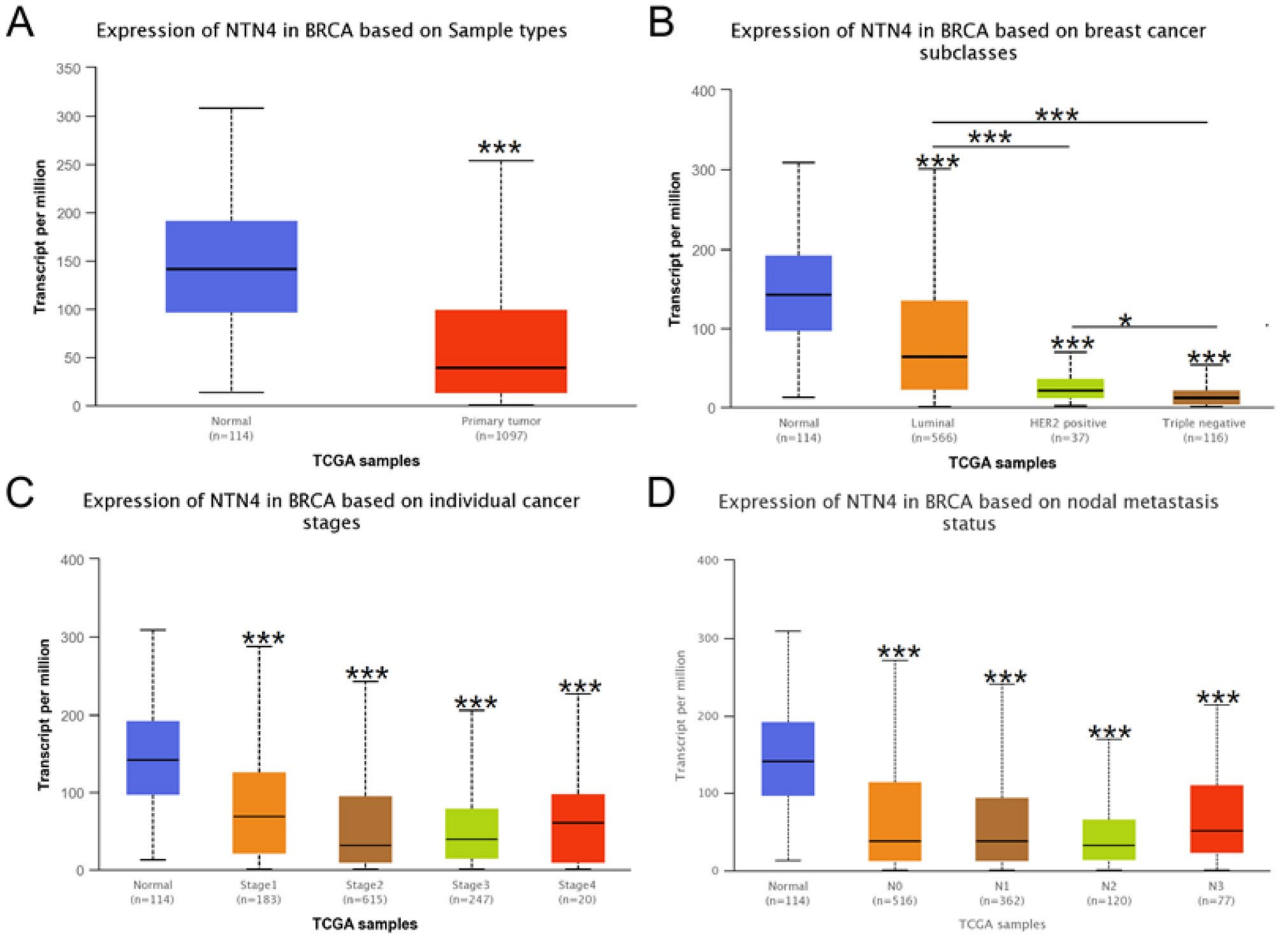
NTN4 mRNA expression in breast cancer based on UALCAN. Expression of NTN4 in breast cancer based on different (**A**) sample types, (**B**) molecular subtypes (in particular TNBC), (**C**) individual clinical stage, (**D**) lymph nodal status.

NTN4 mRNA expression level in breast cancer was explored using UALCAN database. Consistently, NTN4 mRNA expression level in BC tumor was significantly higher than in normal tissue (P < 0.001, Fig. 2A). In different molecular subtypes, luminal had higher mRNA expression of NTN4 than HER2^+^ and triple negative breast cancer (TNBC) (P < 0.001, Fig. 2B). Based on clinical stages, normal tissues had higher NTN4 mRNA expression than all stages of BC (Fig. 2C). In addition, four different stages of lymph node involvement had lower NTN4 mRNA expression than normal tissue (Fig. 2D).

### Relationships between NTN4 promoter methylation and clinicopathological characteristics

**Figure 3:**
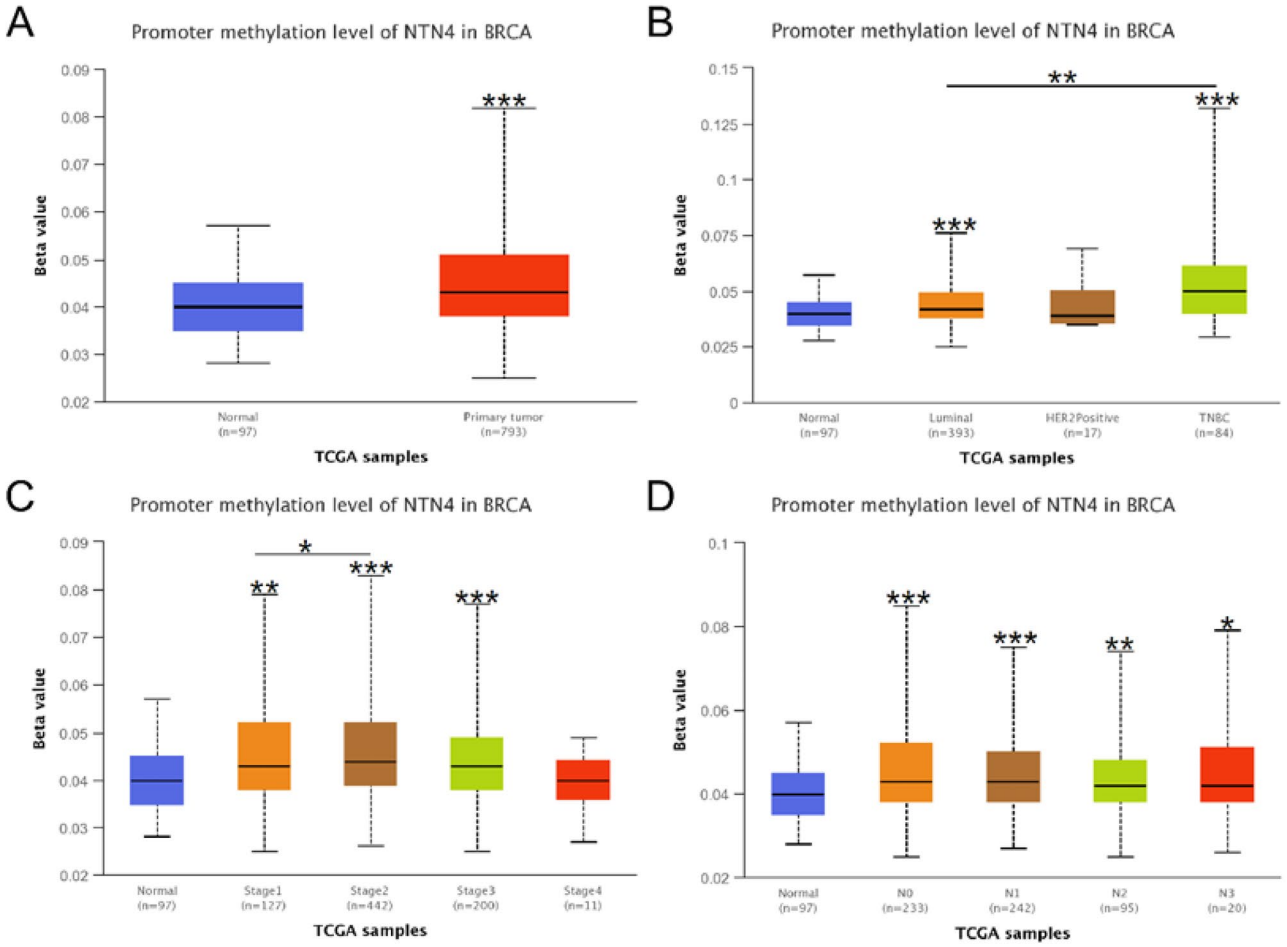
UALCAN analysis of NTN4 promoter methylation in breast cancer. NTN4 promoter methylation levels in breast cancer were compared based on different (**A**) sample types, (**B**) molecular subtypes (TNBC), (**C**) individual cancer stages, (**D**) lymph nodal status.

Using UALCAN database, we explored if promoter methylation of NTN4 was related to clinicopathological characteristics of breast cancer patients. NTN4 promoter methylation level was significantly higher in primary tumor than in normal tissue (P < 0.001, Fig. 3A). Based on molecular subtypes, luminal and TNBC had higher levels of NTN4 promoter methylation (P < 0.001, Fig. 3B). Based on clinical stages, stage 2 and stage 3 had higher levels of NTN4 promoter methylation than stage 4 (Fig. 3C). Furthermore, based on lymph node status, N0 and N1 had higher levels of NTN4 promoter methylation than N3 (Fig. 3D). Thus, NTN4 promoter methylation may contribute to breast cancer development and progression.

### NTN4 mRNA level predicts prognosis in breast cancer

**Figure 4:**
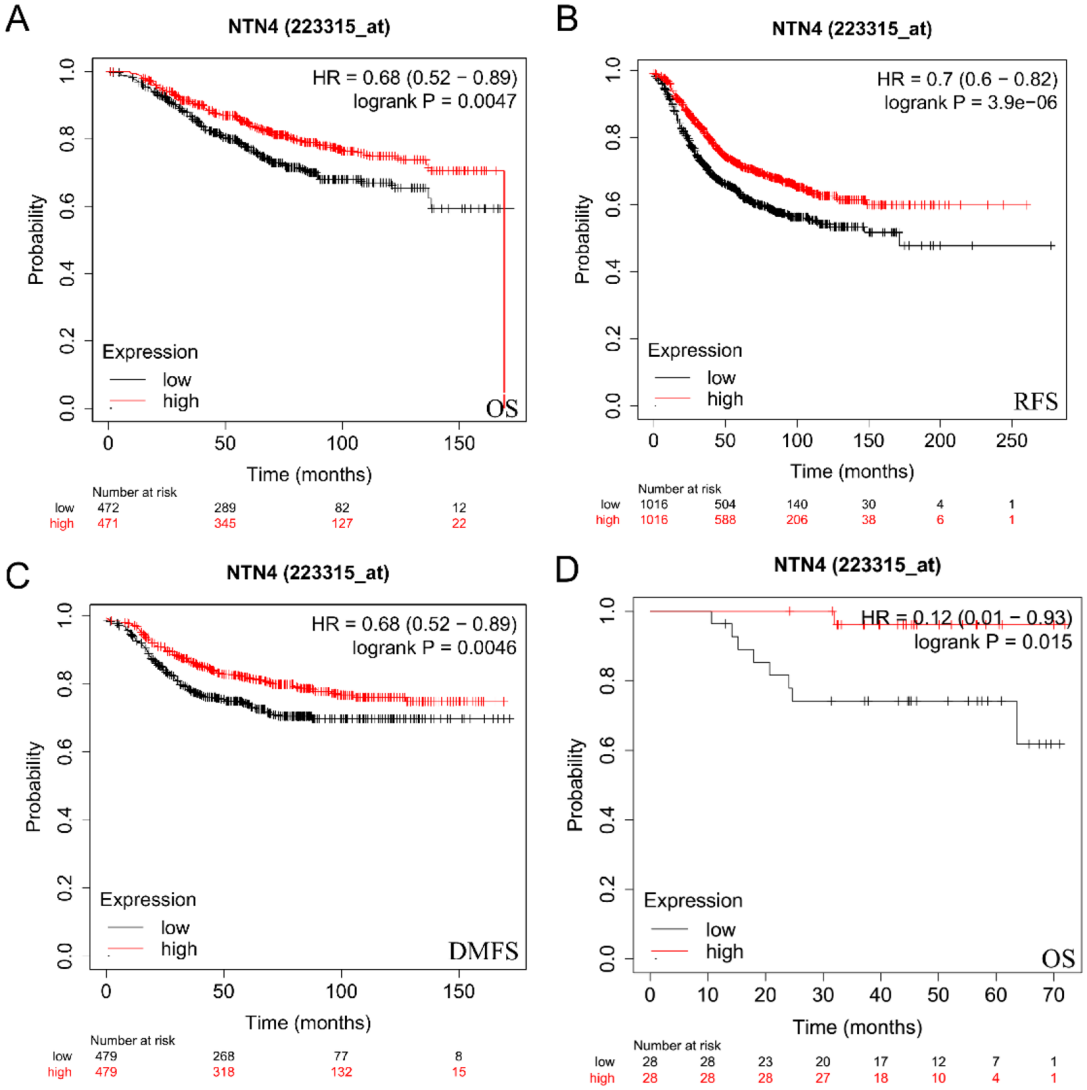
Kaplan–Meier survival curves compare high vs. low expression of NTN4 in breast cancer. High expression of NTN4 was associated with good survival. (**A**) OS: HR (95% CI) 0.68 (0.52–0.89), P = 0.0047. (**B**) RFS: HR (95% CI) 0.7 (0.67–0.82), P = 3.9e−06. (**C**) DMFS: HR (95% CI) 0.68 (0.52–0.89), P = 0.0046. (**D**) Overall survival in TP53 mutant population stratified by NTN4 expression levels, OS: HR (95% CI): 0.12 (0.01–0.93), P = 0.015.

Survival analysis of NTN4 mRNA expression was evaluated using PrognoScan (Supplementary Table 1). Among four cohorts (GSE6532-GPL570, GSE1379, GSE3494-GPL97, GSE4922-GPL97) including different stages of breast cancer, high NTN4 expression was associated with favorable prognosis (Table 2). Similar trend was observed, in Kaplan–Meier plotter database, based on Affymetrix microarrays. Notably, NTN4 significantly correlates with clinical outcome of breast cancer patients, including overall survival (OS), relapse-free survival (RFS), and distant metastasis-free survival (DMFS) (Fig. 4A–C, OS: HR (95% CI) 0.68 (0.52–0.89), P = 0.0047; RFS: HR (95% CI) 0.7 (0.67–0.82), P = 3.9e−06; DMFS: HR (95% CI) 0.68 (0.52–0.89), P = 0.0046, respectively). TP53 is a common mutation gene in breast cancer, and currently no drugs targeted TP53 are available. Therefore, we hypothesized that NTN4 modifies the prognosis in TP53 mutant subpopulation. This may be beneficial to improve the treatment strategy for patients with TP53 mutation. Interestingly, NTN4 significantly affected the OS of TP53 mutant patients (Fig. 4D, OS: HR (95% CI) 0.12 (0.01–0.93), P = 0.015). Therefore, it is conceivable that low NTN4 expression might be a risk factor for a poor prognosis in breast cancer patients.

**Table 2:** Survival analysis of NTN4 mRNA in breast cancer patients (the Prognoscan).

Dataset	Endpoint	Number	ln(HR_high_/HR_low_)	COX P-value	ln(HR)	HR[95% CI^low^ CI^upp^]
GSE6532-GPL570	Relapse Free Survival	87	− 1.54	0.008389	− 0.23	0.79[0.66–0.94]
	Distant Metastasis Free Survival	87	− 1.54	0.008389	− 0.23	0.79[0.66–0.94]
GSE1379	Relapse Free Survival	60	− 1.64	0.033113	− 0.27	0.76[0.59–0.98]
GSE3494-GPL97	Distant Specific Survival	236	− 1.15	0.004196	− 0.38	0.68[0.52–0.89]
GSE4922-GPL97	Distant Free Survival	249	− 0.97	0.043828	− 0.21	0.81[0.66–0.99]

### Correlation between NTN4 mRNA expression and six types of infiltrating immune cells

**Figure 5:**
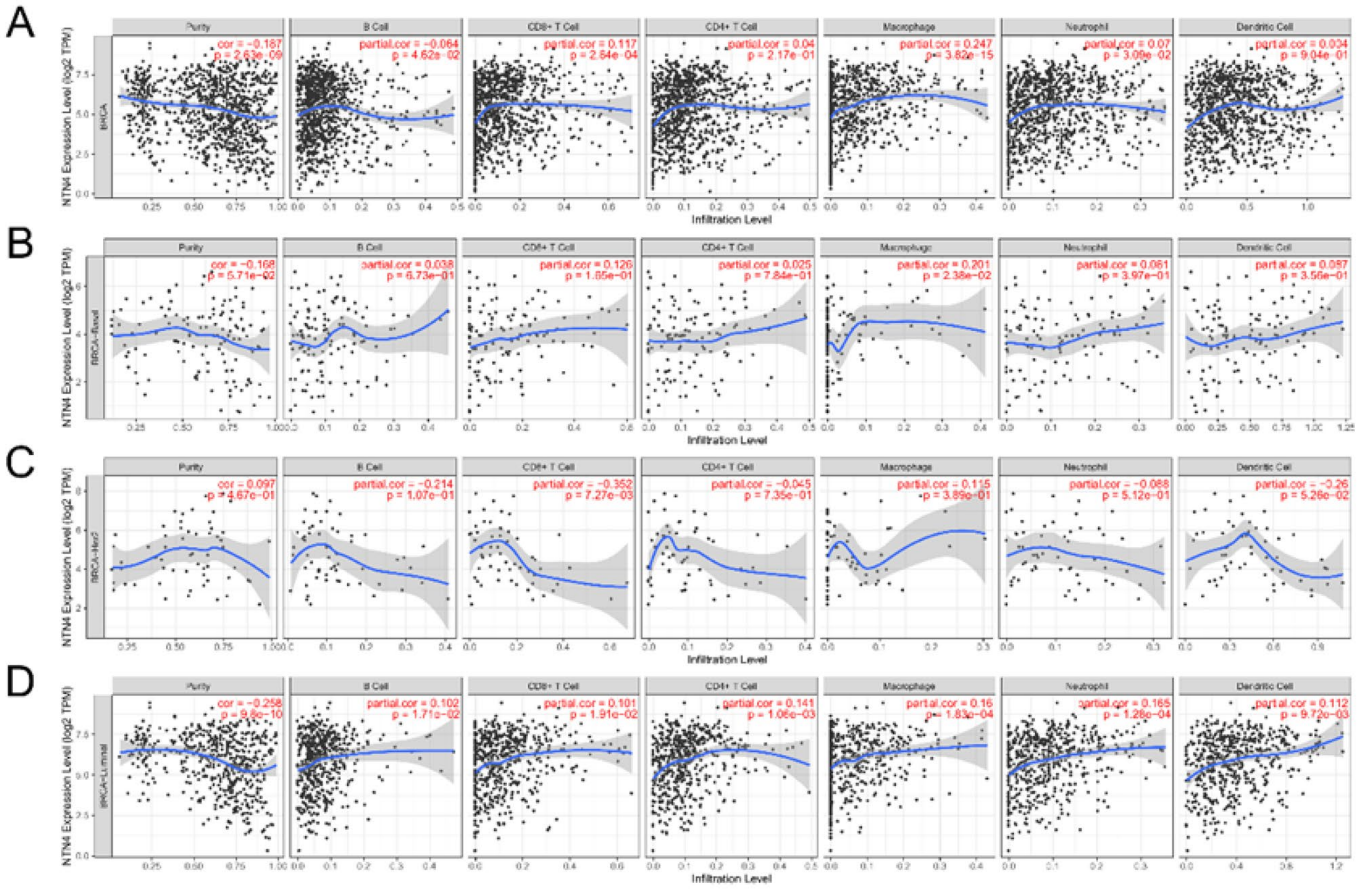
Correlation of NTN4 mRNA expression with immune infiltration level using the TIMER database. (**A**) NTN4 expression was associated with infiltration of CD8^+^ T cells, CD4^+^ T cells, macrophages, neutrophils, or DC in breast cancer. (**B**) Basal subtype, (**C**) Her2^+^ subtype, and (**D**) luminal subtype.

Immune cells in the TME can affect a patient’s survival. Hence, it would be meaningful to explore the association between immune infiltration and NTN4 mRNA expression. We determined if NTN4 mRNA expression was related to immune infiltration in different cancers by calculating coefficient index of NTN4 mRNA expression with immune infiltration in breast cancer using the TIMER. Six types of infiltrating immune cells (B cells, CD4^+^ T cells, CD8^+^ T cells, neutrophils, macrophages, and DC) were explored. NTN4 expression was positively associated with CD8^+^ T cells (r = 0.117, P = 2.64e−04), macrophages (r = 0.247, P = 3.82e−15) and neutrophils (r = 0.07, P = 3.09e−02) in breast cancer whereas negatively with B cells (r = − 0.064, P = 4.62e−02) and tumor purity (r = − 0.187, P = 2.53e−09), but not DC (r = 0.004, P = 9.04e−01) (Fig. 5A). In different breast cancer subtypes, associations differed (Fig. 5B–D). In base-like subtype, NTN4 expression was not related to tumor purity (r = − 0.168, P = 5.71e−02), whereas related to macrophages only (r = 0.201, P = 2.38e−02). In HER2^+^ breast cancer, NTN4 expression level was not related to tumor purity (r = − 0.097, P = 1.67e−01), whereas only negatively related to CD8^+^ T cells (r = − 0.352, P = 7.27e−03). In luminal subtype, NTN4 expression level was negatively associated with tumor purity (r = − 0.258, P = 9.80e−10), whereas positively associated with B cells, CD8^+^ T cells, CD4^+^ T cells, macrophages, neutrophils, and DC.

### Correlation of NTN4 mRNA expression with markers of immune cells

The potential relationship of NTN4 mRNA expression with infiltrating immune cells was explored using the TIMER. Immune cells characterized by cellular markers were deciphered, including B cells, CD8^+^ T cells, M1/M2 macrophages, tumor-associated macrophages (TAM), monocytes, natural killer cell (NK), neutrophils, and DC. Different functional T cells such as Tfh, Th1, Th2, Th9, Th17, Th22, Treg, and exhausted T cells were analyzed (Table 3). In the TIMER, NTN4 mRNA expression levels were significantly related to 33 out of 45 immune cell markers after adjustment for tumor purity.

**Table 3:** Correlations between NTN4 and gene markers of immune cells.

Cell type	Gene marker	None		Purity	
		Cor	P	Cor	P
B cell	CD19	− 0.076	1.17e−02*	− 0.195	5.45e−10***
	CD20	0.011	7.05e−01	− 0.096	2.56e−03**
	CD38	− 0.152	4.25e−07***	− 0.262	3.98e−17***
CD8^+^ T cell	CD8A	0	1e + 00	− 0.122	1.08e−04***
	CD8B	− 0.04	1.87e−01	− 0.153	1.27e−06***
Tfh cell	CXCR5	0.013	6.7e−01	− 0.095	2.73e−03**
	ICOS	− 0.109	3.12e−04***	− 0.226	5.61e−13***
	BCL-6	0.322	5.55e−28***	0.309	2.31e−23***
Th1 cell	IL12RB2	− 0.235	2.98e−15***	− 0.292	4.65e−21***
	WSX-1	0.027	3.67e−01	− 0.049	1.26e−01
	T-BET	− 0.05	9.77e−02	− 0.189	1.81e−09***
Th2 cell	CCR3	0.058	5.32e−02	0.004	9.05e−01
	STAT6	0.355	4.21e−34***	0.333	3.18e−27***
	GATA3	0.332	1.12e−29***	0.396	1.14e−38***
Th9 cell	TGFBR2	0.388	9e−41***	0.351	3.97e−30***
	IRF4	− 0.032	2.86e−01	− 0.16	4.30e−07***
	PU.1	0.033	2.71e−01	− 0.06	3.86e−02*
Th17 cell	IL-21R	0.001	9.67e−01	− 0.114	3.16e−04***
	IL-23R	0.051	9.21e−02	− 0.012	7.08e−01
	STAT3	0.436	3.96e−52***	0.415	1.10e−42***
Th22 cell	CCR10	0.001	9.69e−01	− 0.051	1.09e−01
	AHR	0.475	4.58e−63***	0.432	1.93e−46***
Treg cell	FOXP3	− 0.085	4.97e−03**	− 0.179	1.38e−08***
	CCR8	− 0.069	2.12e−02*	− 0.135	1.88e−05***
	CD25	− 0.165	3.8e−08***	− 0.273	1.86e−18***
T cell exhaustion	PD-1	− 0.122	5.08e−05***	− 0.261	6.15e−17***
	CTLA4	− 0.16	9.97e−08***	− 0.283	8.12e−20***
Macrophage	CD68	0.03	3.22e−01	− 0.044	1.70e−01
	CD11b	0.255	7.54e−18***	0.191	1.28e−09***
M1	NOS2	− 0.013	6.69e−01	− 0.014	6.68e−01
	ROS1	− 0.068	2.34e−02*	− 0.084	8.35e−03**
M2	ARG1	0.016	6.02e−01	−0.005	8.80e−01
	MRC1	0.067	2.68e−02*	− 0.023	4.71e−01
TAM	HLA-G	− 0.036	2.27e−01	− 0.103	1.09e−03**
	CD80	− 0.025	4.16e−01	− 0.093	3.43e−03**
Monocyte	CD14	− 0.001	9.63e−01	− 0.079	1.32e−02*
	CD16	0.103	6.21e−04***	0.045	1.53e−01
NK	XCL1	− 0.021	6.86e−01	− 0.12	1.46e−04***
	KIR3DL1	− 0.052	8.35e−02	− 0.13	4.11e−05***
	CD7	− 0.127	2.36e−05***	− 0.27	4.58e−18***
Neutrophil	CD15	0.069	2.24e−02*	− 0.012	6.98e−01
	MPO	0.023	4.38e−01	− 0.023	4.75e−01
DC	CD1C	0.235	3.29e−15***	0.16	4.03e−07***
	CD141	0.415	4e−47***	0.381	1.21e−35***

### Gene alterations in NTN4 in breast cancer tissue from cBioPortal

**Figure 6:**
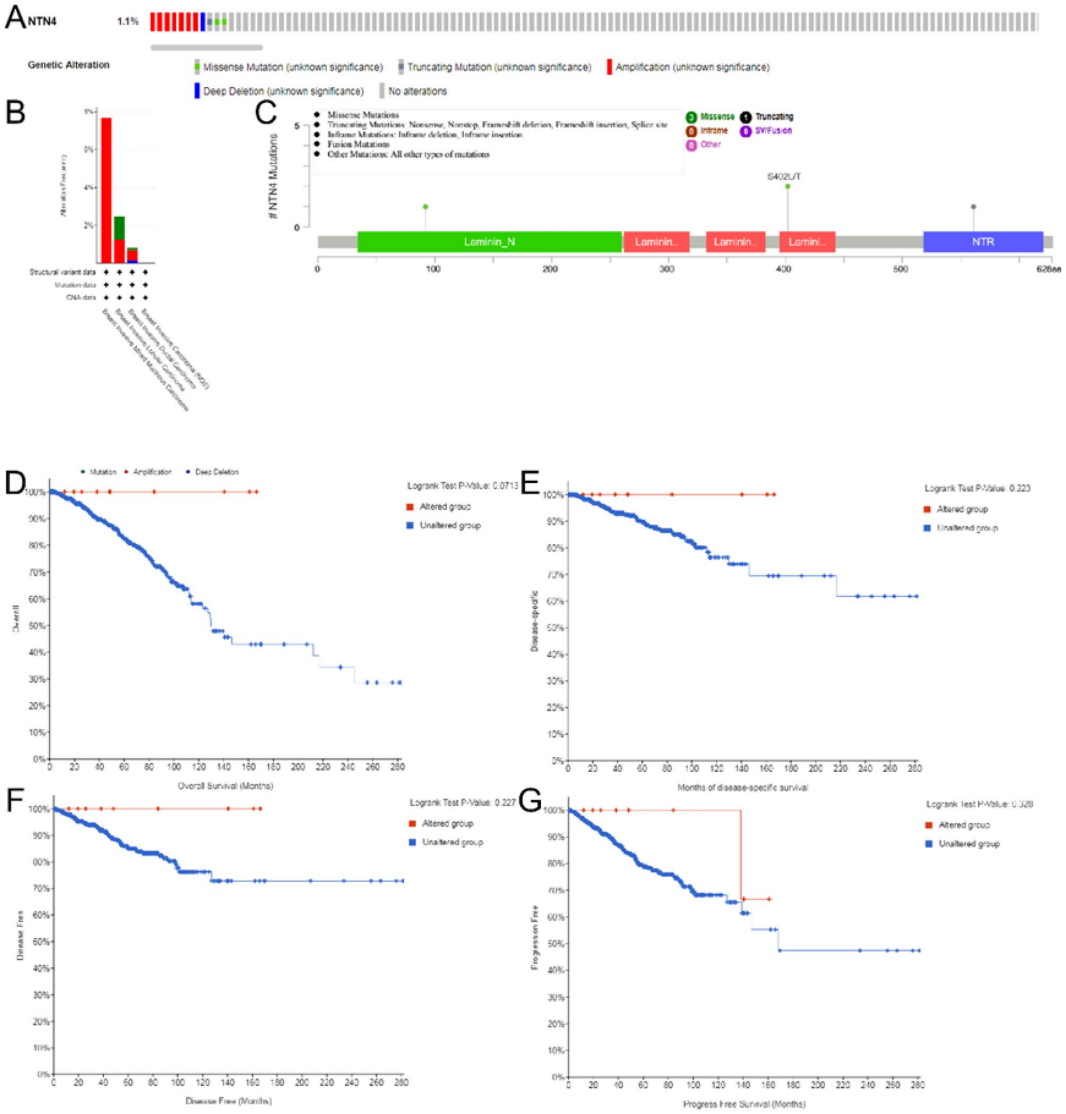
Gene alterations in NTN4 in breast cancer tissue. (**A**) Mutations in NTN4 in breast cancer tissues are shown. (**B**) Frequency of gene alterations in NTN4 in different types of breast cancer. (**C**) Various mutations in breast cancer. (**D**) Kaplan–Meier survival curves analyzed the relationship of gene alterations in NTN4 with overall survival, (**E**) the disease specific survival, (**F**) disease free survival or (**G**) progress free survival of breast cancer patients.

Gene alterations in NTN4 were harbored in 1.1% of sequenced cases from OncoPrint schematic of cBioPortal (Fig. 6A). Among invasive breast carcinoma, no alteration in NTN4 was identified. Among Breast Invasive Ductal Carcinoma, amplification in NTN4 was common. Mutations and deep deletion occurred with an equal frequency. Among Breast Invasive Lobular Carcinoma, amplification and mutation of NTN4 occurred with an equal frequency as well. Amplification of NTN4 occurred in Breast Invasive Mixed Mucinous Carcinoma (Fig. 6B). All mutations of NTN4 in breast cancer were described in Fig. 6C. NTN4 harbored one truncating mutation and three missense mutations. Furthermore, relationship of NTN4 gene alteration with breast cancer patient survival was assessed. However, there was no significant relationship of gene alterations in NTN4 with OS, disease specific survival (DSS), disease free survival (DFS) and progress free survival (PFS) of breast cancer patients (Fig. 6D–G).

### Exploration of NTN4 molecular functions and regulation pathways based on bioinformation tools

**Figure 7:**
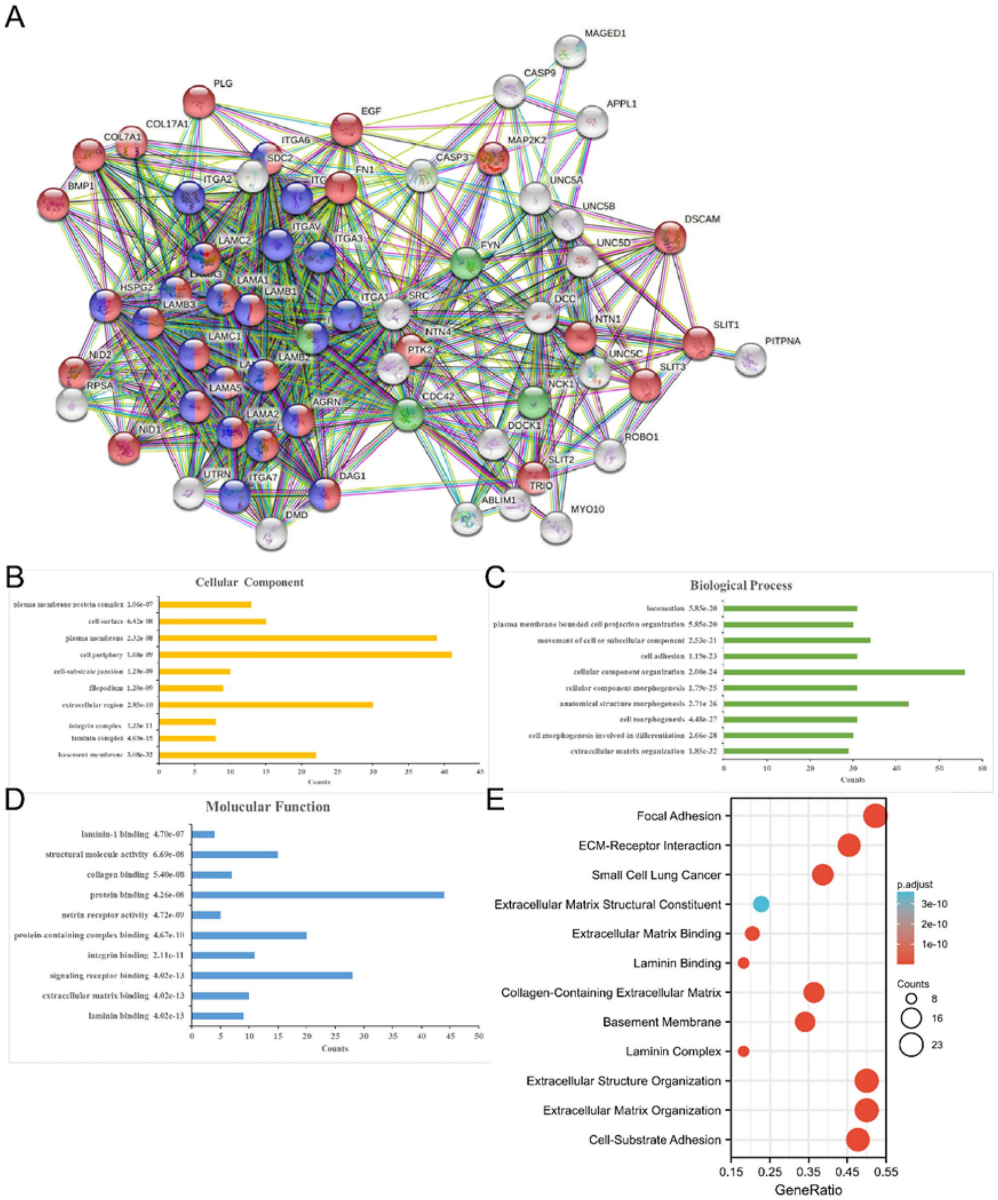
Exploration of NTN4 molecular functions and regulation pathways based on bioinformation tools. (**A**) Interaction network of NTN4 based on the STRING database. (**B**) Cellular component. (**C**) GO biological process. (**D**) Molecular function. (**E**) Enrichment analysis based on GO and KEGG.

Potential molecular function and regulation pathway of NTN4 were preliminarily explored to demonstrate the potential mechanism underlying how NTN4 regulates biological behaviors of breast cancer. First, the STRING database was searched for genes that possibly interact with NTN4 (Fig. 7A). These selected genes were subjected to GO analysis to identify cellular component (CC) (Fig. 7B), biological process (BP) (Fig. 7C) and molecular function (MF) (Fig. 7D) in which NTN4 interacted genes were involved. Based on CC, differentially expressed proteins were extrinsic components of membrane. According to BP, differentially expressed proteins were mainly involved in morphogenesis and motility. Based on MF, differentially expressed proteins functioned mainly for signaling receptor binding. GO and KEGG pathway analysis was performed to identify molecular pathways in which NTN4 interacted genes were involved. The top 12 of enrichment analysis, such as extracellular matrix (ECM)-receptor interaction, adhesion and extracellular part, were presented in Fig. 7E.

## Discussion

Based on data from public databases, NTN4 correlates with breast cancer prognosis and immune infiltration. The NTN4 mRNA expression was significantly lower in invasive breast carcinoma compared with adjacent tissues, while increasing NTN4 mRNA levels are related to favorable prognosis in breast cancer patients.

The NTN4 has been proposed as a prognostic marker. In invasive breast carcinoma, NTN4 expression is associated with longer DFS and OS, as an independent prognostic factor affecting survival^13^. In addition, dysregulated NTN4 has been identified as a potential mediator of breast cancer risk^24^. For example, rs61938093 credible causal variants (CCV) is located in the enhancer that interacts with NTN4 promoter. This risk allele might correlate with reduced activity of NTN4 promoter. Knockout of NTN4 in breast epithelium increased cell proliferation in vitro and tumor growth in vivo, suggesting that low expression of NTN4 promoted breast cancer development. In addition, NTN4 is associated with breast cancer cell migration and invasion via regulation of epithelial mesenchymal transition (EMT)-related genes^25^.

Another important aspect of this study is that NTN4 correlates with diverse immune infiltration (Fig. 5). The NTN4 mRNA levels may reflect infiltration of lymphocytes in breast cancer. In the era of precision medicine^26^, immunological biomarkers are critical^27^ for patient subpopulation selection. Immune biomarkers are numerous^28^, and immune checkpoint inhibitors (ICIs) and tumor mutation burden (TMB) hold promise as such biomarkers^29,30^.

In addition, the frequency of NTN4 gene alteration was low (1.1%), with patterns stratified by molecular subtypes of breast cancer. The NTN4 gene alterations include missense mutation and truncating mutation. However, genetic variation may not affect a patient's survival. Finally, in NTN4 interaction gene cluster analysis, signaling pathways were mostly enriched in cell morphogenesis and motility, which may explain the potential involvement of NTN4 in tumor development and progression of breast cancer.

In this study, for the first time, the relationship of NTN4 with the TME in breast cancer was indicated by bioinformatics. In addition, this study confirmed that NTN4 may be used as a prognostic marker of breast cancer by analyzing a series of clinical samples from patients with in breast cancer. As our findings were obtained from public databases, there are some limitations. With the update of databases, the relationship of NTN4 with prognosis can change accordingly. Similarly, the relationship of NTN4 mRNA level with different immune cell types and markers based on sequencing data from public databases can also change. On the other hand, with accumulation of resources, data stratification will become more refined so that reliability of results may increase. However, further experimental verification is required to validate our findings.

## Conclusion

The current research has explored NTN4 and its prognostic significance in breast cancer. In general, NTN4 is downregulated in breast cancer tissues. Besides, NTN4 is associated with immune infiltration and survival in breast cancer. Collectively, these data suggest that NTN4 is worthy of further investigation in breast cancer, and it may be a potential biomarker, which can be used to predict the prognosis of breast cancer patients.

## Supplementary Information

Supplementary Table S1.

## Data Availability

The datasets of the study have mainly been collected, obtained, and analyzed from corresponding online databases, and corresponding website links were showed in Materials and Methods. The TCGA link website is https://portal.gdc.cancer.gov/exploration?facetTab=cases.The case ID of RNA data from TCGA was as followed: TCGA-AR-A2LE, TCGA-A2-A0ET, TCGA-LL-A7SZ, TCGA-5L-AAT0. TIMER: https://cistrome.shinyapps.io/timer/, UALCAN: ualcan.path.uab.edu/index.html, Kaplan–Meier Plotter: http://www.kmplot.com, PrognoScan; http://dna00.bio.kyutech.ac.jp/PrognoScan/, cBioPortal: http://www.cbioportal.org, String: https://cn.string-db.org/cgi/network?taskId=bsL1tXI2yNb4&sessionId=b7FzfW5U0kqB.
