# Supplementary material for: NTN4 as a prognostic marker and a hallmark for immune infiltration in breast cancer
Source: Sci Rep. 2022 Jun 22;12:10567. doi: 10.1038/s41598-022-14575-2 (PMC9217917; doi:10.1038/s41598-022-14575-2)
Supplement: Supplementary file 1 — Supplementary Table S1. [file 41598_2022_14575_MOESM1_ESM.doc]

**Supplementary Table 1** **Survival analysis of NTN4 mRNA in PrognoScan**

| **Dataset** | **Cancer Type** | **Endpoint** | **N** | **ln(HR-high/**  **HR-low** | **COX**  **P-VALUE** | **ln(HR)** | **HR [95% CI-low CI-upp]** |
| --- | --- | --- | --- | --- | --- | --- | --- |
| GSE13507 | Bladder cancer | Overall Survival | 165 | -0.551396 | 0.292401 | -0.159949 | 0.85 [0.63 - 1.15] |
| GSE13507 | Bladder cancer | Disease Specific Survival | 165 | -1.34491 | 0.00957345 | -0.662803 | 0.52 [0.31 - 0.85] |
| GSE12417-GPL97 | Blood cancer | Overall Survival | 163 | -0.469137 | 0.0561232 | -1.52293 | 0.22 [0.05 - 1.04] |
| GSE12417-GPL570 | Blood cancer | Overall Survival | 79 | 0.556922 | 0.253608 | 0.843688 | 2.32 [0.55 - 9.90] |
| GSE16131-GPL97 | Blood cancer | Overall Survival | 180 | 0.578752 | 0.363696 | -0.100179 | 0.90 [0.73 - 1.12] |
| GSE2658 | Blood cancer | Disease Specific  Survival | 559 | -0.66206 | 0.164286 | -0.15839 | 0.85 [0.68 - 1.07] |
| GSE4271-GPL97 | Brain cancer | Overall Survival | 77 | -1.09505 | 0.00283153 | -0.316312 | 0.73 [0.59 - 0.90] |
| GSE7696 | Brain cancer | Overall Survival | 70 | -1.14594 | 0.0171699 | -0.288884 | 0.75 [0.59 - 0.95] |
| GSE4412-GPL97 | Brain cancer | Overall Survival | 74 | -1.18386 | 0.0104469 | -0.41157 | 0.66 [0.48 - 0.91] |
| GSE16581 | Brain cancer | Overall Survival | 67 | -1.02894 | 0.410067 | -0.315721 | 0.73 [0.34 - 1.55] |
| GSE19615 | Breast cancer | Distant Metastasis Free Survival | 115 | -0.458495 | 0.701737 | -0.0916472 | 0.91 [0.57 - 1.46] |
| GSE12276 | Breast cancer | Relapse Free Survival | 204 | -0.403894 | 0.351059 | -0.0527622 | 0.95 [0.85 - 1.06] |
| GSE6532-GPL570 | Breast cancer | Relapse Free Survival | 87 | -1.53652 | 0.00838898 | -0.234346 | 0.79 [0.66 - 0.94] |
| GSE6532-GPL570 | Breast cancer | Distant Metastasis Free Survival | 87 | -1.53652 | 0.00838898 | -0.234346 | 0.79 [0.66 - 0.94] |
| GSE9195 | Breast cancer | Relapse Free Survival | 77 | -15.3898 | 0.427682 | -0.127785 | 0.88 [0.64 - 1.21] |
| GSE9195 | Breast cancer | Distant Metastasis Free Survival | 77 | -1.26896 | 0.310652 | -0.183389 | 0.83 [0.58 - 1.19] |
| GSE1378 | Breast cancer | Relapse Free Survival | 60 | -0.830609 | 0.134573 | -0.164258 | 0.85 [0.68 - 1.05] |
| GSE1379 | Breast cancer | Relapse Free Survival | 60 | -1.6414 | 0.0331129 | -0.270926 | 0.76 [0.59 - 0.98] |
| GSE9893 | Breast cancer | Overall Survival | 155 | -1.09607 | 0.381197 | -0.116201 | 0.89 [0.69 - 1.15] |
| GSE1456-GPL97 | Breast cancer | Overall Survival | 159 | -0.987847 | 0.164941 | -0.236047 | 0.79 [0.57 - 1.10] |
| GSE1456-GPL97 | Breast cancer | Relapse Free Survival | 159 | -0.681313 | 0.213883 | -0.212549 | 0.81 [0.58 - 1.13] |
| GSE1456-GPL97 | Breast cancer | Disease Specific Survival | 159 | -0.982758 | 0.112788 | -0.315534 | 0.73 [0.49 - 1.08] |
| GSE3494-GPL97 | Breast cancer | Disease Specific Survival | 236 | -1.14908 | 0.00419592 | -0.384724 | 0.68 [0.52 - 0.89] |
| GSE4922-GPL97 | Breast cancer | Disease Free Survival | 249 | -0.971711 | 0.0438276 | -0.214093 | 0.81 [0.66 - 0.99] |
| GSE17536 | Colorectal cancer | Overall Survival | 177 | 0.0380805 | 0.26139 | 0.169001 | 1.18 [0.88 - 1.59] |
| SE17536 | Colorectal cancer | Disease Specific Survival | 177 | 0.269714 | 0.595954 | 0.0927664 | 1.10 [0.78 - 1.55] |
| GSE17536 | Colorectal cancer | Disease Free Survival | 145 | 0.367755 | 0.761556 | 0.0651329 | 1.07 [0.70 1.63] |
| GSE14333 | Colorectal cancer | Disease Free Survival | 226 | 0.0720096 | 0.149646 | 0.265519 | 1.30 [0.91 - 1.87] |
| GSE17537 | Colorectal cancer | Disease Free Survival | 55 | 0.168557 | 0.941835 | -0.0234614 | 0.98 [0.52 - 1.83] |
| GSE17537 | Colorectal cancer | Disease Specific Survival | 49 | 0.353456 | 0.509284 | 0.25962 | 1.30 [0.60 - 2.80] |
| GSE17537 | Colorectal cancer | Overall Survival | 55 | 0.116919 | 0.251917 | 0.320298 | 1.38 [0.80 - 2.38] |
| GSE11595 | Esophagus cancer | Overall Survival | 34 | 1.20641 | 0.302152 | 0.440764 | 1.55 [0.67 - 3.59] |
| GSE22138 | Eye cancer | Distant Metastasis Free Survival | 63 | -0.653728 | 0.612698 | -0.0654836 | 0.94 [0.73 - 1.21] |
| GSE2837 | Head and neck cancer | Relapse Free Survival | 28 | -0.912604 | 0.861567 | 0.215789 | 1.24 [0.11 - 14.03] |
| GSE2837 | Head and neck cancer | Relapse Free Survival | 28 | 0.913378 | 0.870553 | -0.284372 | 0.75 [0.02 - 23.01] |
| GSE13213 | Lung cancer | Overall Survival | 117 | 0.297116 | 0.510498 | -0.0862231 | 0.92 [0.71 - 1.19] |
| GSE31210 | Lung cancer | Overall Survival | 204 | -1.30249 | 0.0286209 | -0.603966 | 0.55 [0.32 - 0.94] |
| GSE31210 | Lung cancer | Relapse Free Survival | 204 | -1.25295 | 0.0107074 | -0.535195 | 0.59 [0.39 - 0.88] |
| GSE11117 | Lung cancer | Overall Survival | 41 | 0.472152 | 0.88913 | 0.0295577 | 1.03 [0.68 - 1.56] |
| GSE3141 | Lung cancer | Overall Survival | 111 | -0.912694 | 0.600513 | -0.0921472 | 0.91 [0.65 - 1.29] |
| GSE4716-GPL3694 | Lung cancer | Overall Survival | 50 | -1.22523 | 0.0181558 | -1.91246 | 0.15 [0.03 - 0.72] |
| GSE8894 | Lung cancer | Relapse Free Survival | 138 | 0.502283 | 0.32112 | 0.079799 | 1.08 [0.93 - 1.27] |
| GSE17710 | Lung cancer | Relapse Free Survival | 56 | -0.866605 | 0.543223 | -0.0906407 | 0.91 [0.68 - 1.22] |
| GSE17710 | Lung cancer | Overall Survival | 56 | -0.669318 | 0.559395 | -0.0903438 | 0.91 [0.67 - 1.24] |
| GSE17710 | Lung cancer | Relapse Free Survival | 56 | -1.02016 | 0.378354 | -0.129461 | 0.88 [0.66 - 1.17] |
| GSE17710 | Lung cancer | Overall Survival | 56 | -0.785874 | 0.554451 | -0.090991 | 0.91 [0.68 - 1.23] |
| GSE17710 | Lung cancer | Relapse Free Survival | 56 | -0.435002 | 0.645226 | -0.0788899 | 0.92 [0.66 - 1.29] |
| SE17710 | Lung cancer | Overall Survival | 56 | -1.08745 | 0.385842 | -0.132467 | 0.88 [0.65 - 1.18] |
| GSE17710 | Lung cancer | Relapse Free Survival | 56 | -0.554504 | 0.554614 | -0.0876173 | 0.92 [0.69 - 1.23] |
| GSE17710 | Lung cancer | Overall Survival | 56 | -0.537451 | 0.636118 | -0.0843048 | 0.92 [0.65 - 1.30] |
| GSE9891 | Ovarian cancer | Overall Survival | 278 | 0.182838 | 0.883452 | -0.0100737 | 0.99 [0.87 - 1.13] |
| GSE17260 | Ovarian cancer | Progression Free Survival | 110 | 0.0470425 | 0.511776 | -0.0584067 | 0.94 [0.79 - 1.12] |
| GSE17260 | Ovarian cancer | Overall Survival | 110 | 0.0860064 | 0.442537 | 0.0898513 | 1.09 [0.87 - 1.38] |
| GSE19234 | Skin cancer | Overall Survival | 38 | -0.826142 | 0.420391 | -0.172643 | 0.84 [0.55 - 1.28] |
